# A Novel Function of Controlled-Release Nitrogen Fertilizers

**DOI:** 10.1264/jsme2.ME2902rh

**Published:** 2014-07-19

**Authors:** Masahito Hayatsu

**Affiliations:** 1 National Institute for Agro-Environmental Sciences, 3–1–3 Kannondai, Tsukuba, Ibaraki 305–8604, Japan

Nitrogen is the most important essential nutrient that plays a major role in achieving maximum crop yield in agriculture. Therefore, nitrogen fertilizers such as ammonium sulfate and urea have been extensively used in modern agriculture. These fertilizers are generally oxidized to nitrate via nitrite by nitrifying microorganisms in the agricultural field (4, 10, 11). The serious environmental problems associated with the use of nitrogen fertilizers are nitrate contamination of ground and surface water due to nitrate leaching and loss from the agricultural field (15). For these reasons, controlled-release nitrogen fertilizers have been developed to enable a slow release of nitrogen into the soil during the crop-growing season. The use of controlled-release nitrogen fertilizers is mainly based on the principle of nitrogen utilization efficiency for crop production. However, in the current issue, Ikeda *et al.* report that the controlled-release nitrogen fertilizer urea-formaldehyde unexpectedly modifies the microbial community structure in the phytosphere of crops (8).

Controlled-release nitrogen fertilizers consists of three major types: biologically or chemically degradable organic nitrogen, nitrogen coated with a physical barrier, and lowly soluble nitrogen. Controlled-release nitrogen fertilizers have recently received increased attention because of their potential N_2_O emission-reducing properties (1). Nitrous oxide, which is produced by nitrification and denitrification (4, 10, 11), is an effective earth-warming gas with a 298-fold higher efficiency than carbon dioxide. Nitrogen fertilizers such as urea, ammonium sulfate, and ammonium nitrate are sources of N_2_O. Recent studies have revealed that slow-release nitrogen fertilizers reduce N_2_O emissions from the agricultural field (1, 24). The urea-formaldehyde fertilizer, which is a representative controlled-release nitrogen fertilizer, is synthesized from urea and formaldehyde in the presence of a catalyst at various temperature conditions (2).

The urea-formaldehyde fertilizer is degraded by soil microorganisms, resulting in the gradual release of urea into the soil, which is then further metabolized by soil microorganisms into plant available nitrogen forms such as ammonium and nitrate (12, 13). Urea-formaldehyde fertilizers contain short-chain and long-chain methylene urea polymers. The nitrogen release rates of the fertilizer into the soil are dependent on the content ratio of the long-chain polymers. Previous studies on the influence of microorganisms on urea-formaldehyde fertilizers in soils have examined the processes of urea-formaldehyde degradation and urea release rate to evaluate its nutrient efficiency for crop production (2, 12). PCR-denaturing gradient gel electrophoresis (DGGE) analysis has detected differences in bacterial community structure between urea-treated and methylene urea-treated soils (3). *Ochrobactrum anthropi* (14), *Ralstonia paucula* (13), and *Agrobacterium tumefaciens* (17) have been identified as the major microorganisms that degrade urea-formaldehyde fertilizers. These bacteria produce different types of urea-formaldehyde-hydrolyzing enzymes that produce ammonia, urea, and formaldehyde. The urea-formaldehyde-degrading enzyme purified from *O. anthropi* hydrolyzes different lengths of methylene urea oligomers into ammonium, formaldehyde, and urea.

The interface between plants and the external environment is called the phytosphere, which consists of the phylloplane, rhizosphere, and rhizoplane (22). The microbial community in the phytosphere plays important roles in the defense against plant pathogens (16, 20, 25) and environmental stresses, as well as in essential reactions of nutrient dynamics (9, 19). Molecular ecological methods have been recently employed to investigate the community structure of microorganisms in the phytosphere (22). Previous studies by Ikeda and coworkers have shown that tissues (*i.e.*, leaves, stems, roots, and tubers) (26), crop species (23), various environmental factors and agricultural management (5, 6, 27) could negatively or positively affect the diversity and abundance of microorganisms in the phytosphere. For example, nitrogen fertilizer (urea) application levels affected the bacterial community structure in the rhizosphere of rice in paddy fields (7). Low-level nitrogen fertilizers shifted the community structure including important microorganisms for plant associations and methane metabolism in the paddy soil and rice. Additionally, microorganisms in the phytosphere have been reported to improve crop growth and prevent plant pathogen infection (19, 28).

Ikeda *et al.* conducted field experiments to evaluate the effect of urea-formaldehyde fertilizers on the microbial community in the underground tissues of crops. They observed that the application of urea-formaldehyde fertilizers to onion bulbs and the main roots of sugar beet changed the diversity of the microbial community and the abundances of certain bacterial species. This unexpected effect could be thought to be beneficial to crop growth because these bacterial strains were identified as plant growth-promoting bacteria based on the analysis of their 16S rRNA gene sequences. Another interesting result is the increase in the number of bacterial strains that are capable of metabolizing C1 compounds, which are the metabolites of urea-formaldehyde fertilizers. They expected that urea-formaldehyde fertilizers were not only an effective nitrogen source but also a useful driving force for the control of the microbial community structure in the phytosphere. These studies have thus shown that specific fertilization practices (*i.e.*, types of fertilizer and level and frequency of fertilization) are important factors that shape the microbial community structure in the phytosphere.

Nitrogen fertilizers have been increasingly used as a nutrient source for crops consumed by the growing human population. The current global consumption of nitrogen fertilizers is estimated to be approximately 100 million tons per year. Based on these circumstances, controlled-release nitrogen fertilizers are considered a good nitrogen source for crops. Ikeda *et al.* clearly demonstrated that fertilizers played an additional function, which is the improvement of the microbial community in the rhizosphere. Their findings provide fresh insights into the direction of research on agricultural microbial ecology and fertilization management. It is expected that their research will eventually confirm the positive effects and mechanisms associated with the changes in the microbial community caused by the application of nitrogen fertilizers to crops. Recent advances in molecular ecological technologies (18, 30) and sequencing methods (21, 29) will also facilitate a better understanding of these issues. Further studies on the role of fertilizers on the microbial community in the phytosphere could lead to innovative changes in fertilization practices in modern agriculture.
